# Imaging of Functional Brain Circuits during Acquisition and Memory Retrieval in an Aversive Feedback Learning Task: Single Photon Emission Computed Tomography of Regional Cerebral Blood Flow in Freely Behaving Rats

**DOI:** 10.3390/brainsci11050659

**Published:** 2021-05-18

**Authors:** Katharina Braun, Anja Mannewitz, Joerg Bock, Silke Kreitz, Andreas Hess, Henning Scheich, Jürgen Goldschmidt

**Affiliations:** 1 Department of Zoology/Developmental Neurobiology, Institute of Biology, Otto von Guericke University Magdeburg, 39106 Magdeburg, Germany; Anja.Mannewitz@smul.sachsen.de; 2 Center for Behavioral Brain Sciences, 39106 Magdeburg, Germany; joerg.bock@ovgu.de (J.B.); Henning.Scheich@lin-magdeburg.de (H.S.); Juergen.Goldschmidt@lin-magdeburg.de (J.G.); 3 PG “Epigenetics and Structural Plasticity”, Institute of Biology, Otto von Guericke University Magdeburg, 39106 Magdeburg, Germany; 4 Institute of Experimental and Clinical Pharmacology and Toxicology, Friedrich-Alexander University, 91054 Erlangen, Germany; silke.kreitz@fau.de (S.K.); andreas.hess@pharmakologie.uni-erlangen.de (A.H.); 5 Combinatorial Neuroimaging Core Facility, Leibniz Institute for Neurobiology, 39106 Magdeburg, Germany

**Keywords:** functional imaging, avoidance learning, acquisition, memory retrieval, prefrontal cortex, limbic system

## Abstract

Active avoidance learning is a complex form of aversive feedback learning that in humans and other animals is essential for actively coping with unpleasant, aversive, or dangerous situations. Since the functional circuits involved in two-way avoidance (TWA) learning have not yet been entirely identified, the aim of this study was to obtain an overall picture of the brain circuits that are involved in active avoidance learning. In order to obtain a longitudinal assessment of activation patterns in the brain of freely behaving rats during different stages of learning, we applied single-photon emission computed tomography (SPECT). We were able to identify distinct prefrontal cortical, sensory, and limbic circuits that were specifically recruited during the acquisition and retrieval phases of the two-way avoidance learning task.

## 1. Introduction

Active avoidance learning represents an evolutionarily old and quite complex form of aversive feedback learning that is essential for actively coping with unpleasant, aversive or dangerous situations that are frequently encountered in daily life. During training the individual first uses an escape strategy, i.e., it removes itself from the unpleasant situation. During ongoing training the individual then develops a behavioral strategy with which it can avoid the unpleasant situation, i.e., the initial escape response is transferred into an active, goal-directed avoidance reaction. The reinforcing drive during the acquisition of an active avoidance strategy is the absence of the predicted aversive stimulus, i.e., the acquisition of an active avoidance response may be considered as specific Pavlovian instrumental transfer [1,2]. This view is supported by the assumption that successfully avoiding an aversive outcome is rewarding [3], which is also indicated by the release of dopamine in the prefrontal cortex once the animal has “understood” the benefits of avoidance [4,5].

While the neuronal circuitry of Pavlovian fear conditioning has been extensively investigated, less is known about the functional circuits involved in aversive feedback learning. Using the two-way avoidance (TWA) learning paradigm in rats, the aim of this study was to obtain an overall picture of the brain circuits involved in active avoidance learning. We applied single-photon emission computed tomography (SPECT) of cerebral blood flow, an imaging technique that allows a longitudinal assessment of activation patterns in the brain of freely behaving animals during different stages of learning [6]. More specifically, we aimed to identify brain circuits specifically activated during two phases of active avoidance learning, the acquisition phase and the memory retrieval phase.

## 2. Materials and Methods

### Subjects and Housing

Female Wistar rats (*n* = 10 from five different litters) bred and raised in our animal facility were used for the experiments. All animals were housed in translucent standard laboratory cages type IV (Tecniplast Deutschland GmbH, Hohenpeißenberg, Germany) under standard animal facility conditions (temperature: 22 ± 2 °C; humidity: 55 ± 5%; artificial 12 h/12 h light/dark cycle, light on at 06:00 a.m.) with free access to food and water. Cages were cleaned once a week. At the day of birth (P0) litter size was standardized to four female pups per dam. At P21, all pups were weaned from their mother and housed in sibling groups until P67. Ten days prior to the start of the experiment the animals were individually housed in standard rat cages type III (Tecniplast Deutschland GmbH, Hohenpeißenberg, Germany).

All experimental procedures were approved by the Ethics Committee of the government of the state of Saxony-Anhalt, Germany, according to the German guidelines for the care and use of animals in laboratory research (Az.: 42502-2-1008 UniMD).

## 3. Behavioral Experiments

### 3.1. Two-Way Avoidance (TWA) Training

The behavioral experiments were conducted as described in [7], using fully automated shuttle boxes (SB) (Hasomed GmbH, Magdeburg, Germany). Adult rats were trained in a shuttle box of 50 cm × 25.5 cm × 53 cm (length × depth × height) size equipped with a grid floor of 0.6 cm diameter bars spaced 1.95 cm apart. A vertical non-transparent plastic wall bisected the compartments with an opening in the center, allowing the rats to freely move to the opposite compartment. Infrared light beams determined the position of the animal. Shuttle boxes were cleaned after each animal with 70% ethanol (Roth, Karlsruhe, Germany).

**Figure 1:**
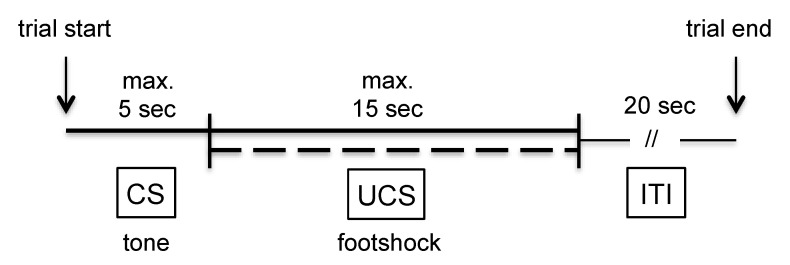
Experimental design for one learning trial. The tone (CS) was presented for 5 s, immediately followed by a 15-s period of foot shocks (CS + UCS). A trial was terminated by a 20-s intertrial interval (ITI), after which the next trial was started.

The animals were trained on four consecutive days. Each training session started with 3 min of habituation allowing the rats to explore the learning environment. Thereafter, the animals were exposed to 50 consecutive learning trials/day using the training procedure outlined in Figure 1: conditioned stimulus (CS) during which a tone of 2.4 kHz frequency and 80 dB loudness was presented for a maximal duration of 5 s. Immediately following the CS the unconditioned stimulus (UCS), a 0.6 mA foot shock, was added for a maximal duration of 15 s (CS + UCS). The following behavioral parameters were recorded and analyzed: number of avoidance reactions, number of escape reactions, number of failures, and escape latency. An avoidance reaction was recorded when the animal moved to the other compartment during CS presentation, an escape reaction was recorded when the animal moved to the other compartment during CS + UCS presentation. Failure was defined as no movement to the other compartment during CS or CS + UCS presentation.

### 3.2. Experimental Groups

Adult female rats (*n* = 10) were exposed to TWA training at P80–P84. Blood flow patterns during the first training day (acquisition) at P80 and during the fifth test day (memory retrieval) at P84 were compared.

### 3.3. Imaging Experiments

#### 3.3.1. Jugular Vein Catheterization

The procedure was conducted as described in detail by [7,8,9]. For implantation of the catheter into the right external jugular vein the rats were anesthetized with 2% isoflurane in a 2:1 O_2_:N_2_O volume ratio. A catheter (11 cm long soft silicon tube, ID 0.5 mm, OD 1.3 mm, Gaudig Laborfachhandel GbR, Magdeburg, Germany) was inserted into the jugular vein of each rat and connected via a 20-G needle to a 1 mL syringe. Before insertion the syringe and the catheter were filled with a heparinized (50 IU/mL) saline (0.9% NaCl) solution (concentration 3:10, heparin:saline). Animals were allowed to recover from jugular vein catheterization for 2–3 days prior to the onset of the experiment. The catheters were flushed every second day with heparinized (50 IU/mL) saline solution (concentration 1:10, heparin:saline) to prevent blood clotting and maintain functionality.

#### 3.3.2. Intravenous ^99m^Tc-HMPAO Application in Freely Behaving Animals

On the day of the learning experiment each jugular vein catheter was connected to an 80 cm long 1/16” teflon tube (CS Chromatographieservice GmbH, Langerwehe, Germany) filled with saline. The blood flow tracer 99m-technetium hexamethyl propylene amine oxime (^99m^Tc-HMPAO) was prepared using commercial kit preparations (Ceretec, GE Healthcare, Buchler, Braunschweig, Germany) as described previously [10]. A rat was placed in the shuttle box for avoidance training. The teflon tube was connected to a swivel system to allow the animal to freely move between the two compartments without being irritated or restrained by the tube. A 23-G needle and 1 mL syringe used for intravenous injection was first filled with approximately 200 MBq ^99m^Tc- HMPAO (volume 400–500 μL) and also connected to the swivel system. An automatic syringe pump (Harvard Apparatus US, Hugo Sachs Elektronik, March, Germany) provided continuous injection of the tracer. The injection was started at the onset of the learning trials and terminated after 5 min.

#### 3.3.3. In Vivo SPECT-Imaging of Regional Cerebral Blood Flow

Immediately after the shuttle-box sessions the animals were anesthetized (4% isoflurane in a 2:1 O_2_:N_2_O volume ratio) and transferred to the SPECT/CT scanner. Imaging was performed using a four-head NanoSPECT/CT scanner (Mediso, Münster, Germany). The animals were SPECT-scanned for two hours under continuous gas anesthesia (1.4% isoflurane in a 2:1 O_2_:N_2_O volume ratio) with nine-pinhole apertures with 1.5 mm pinhole diameters providing a nominal resolution of ≤1.2 mm. Axial field of view of was 38.9 mm. Photopeaks were set to 140 keV ± 5%, the default values of the NanoSPECT/CT. Coregistered CT scans were taken before and after the SPECT scans with the following parameters: 55 kVp, 177 µA, 360 projections, 500 ms per projection. These scans were used as anatomical references and for control of head motion between beginning and end of the imaging session.

#### 3.3.4. SPECT Data Analysis

CT and SPECT images were reconstructed using the manufacturer’s software (CT: InVivoScope 1.43, Bioscan Inc., Washington DC, USA; SPECT: HiSPECT^TM^, SCIVIS, Goettingen, Germany). SPECT images were reconstructed with isotropic voxel sizes of 333 μm, CT- images with 200 μm. SPECT images were aligned to an in-house reference MRI [9] using the coregistered CT [6]. Based on skull landmarks, the data sets from the CT measurements were manually aligned to the MR using the MPI-tool^TM^ software (Multi Purpose Image Tool V6.42, ATV GmbH, Kerpen, Germany). SPECT brain data were cut out of the SPECT head data sets with the OsirixTM software (32-bit version 3.7 and 64-bit version 5.7.1) using a whole-brain volume of interest (VOI) made from the MRT rat brain template.

For illustration of the average blood flow in each group, global-mean normalized SPECT images of all animals in each group were added. Voxel-wise statistics were collected with the MagnAn-software (version 2.4, BioCom GbR, D-90180 Uttenreuth, Germany) [11,12].

To identify significant differences of regional blood flow during the acquisition and retrieval phase the SPECT images obtained for the two different learning paradigms were compared using a voxel-wise paired T-test (VBM analysis). The three-dimensional p-map obtained was thresholded at *p* ≤ 0.05 and at *p* ≤ 0.01, respectively, in order to identify the significantly different voxels. We used the 5% significance level as an exploratory level that, due to larger cluster sizes, can facilitate in serial sections visual identification of brain regions and systems, but we restricted our analysis and conclusions to the 1% significance level. We used uncorrected p-values, a procedure common in small-animal radionuclide imaging [13,14]. Illustrations of the analyzed and fused SPECT/MRI images were compiled using the OsiriX-DICOM viewer.

Brain regions were identified using the purpose-built brain atlas from MagnAn software derived from the rat brain atlas from [15]. The brain regions identified were assigned to five functional systems: sensory/motor cortex, subcortical systems, association/prefrontal cortex, medial temporal lobe (MTL)/limbic system, and limbic output regions.

## 4. Results

**Figure 2:**
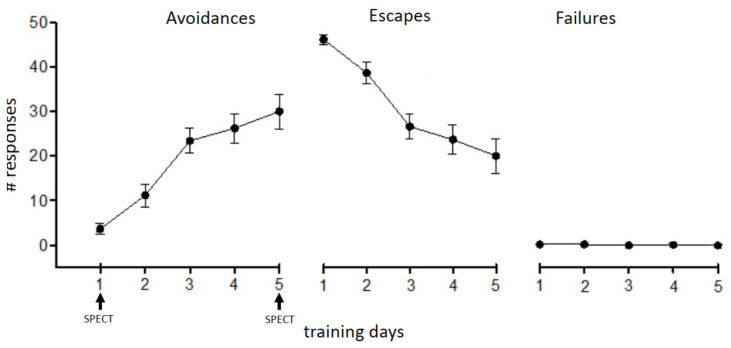
Development of TWA learning. During the first training day the animals mainly showed escape responses; with continuing training the number of avoidance responses increased.

The behavioral results basically confirmed results obtained in previous experiments [16,17,18]. While on the first training day the animals mainly showed escape responses, the number of avoidance responses increased with continuing training (Figure 2).

### 4.1. Comparing Regional Cerebral Blood Flow during Acquisition and Retrieval

**Figure 3:**
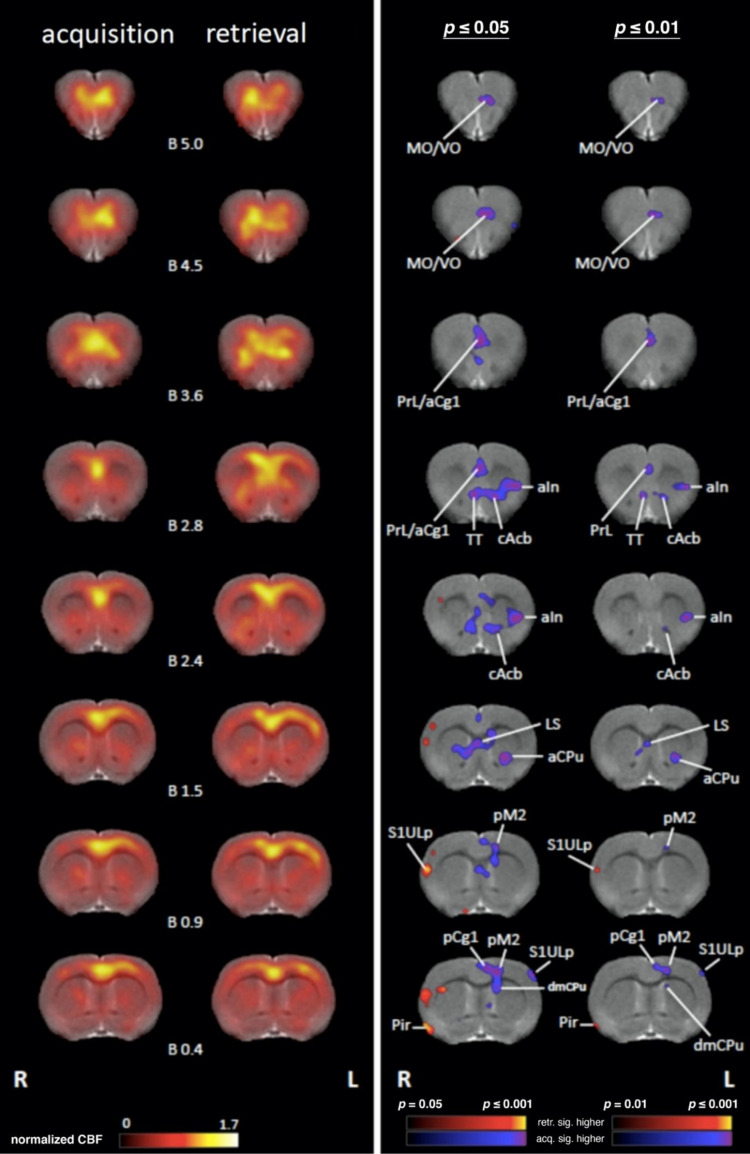

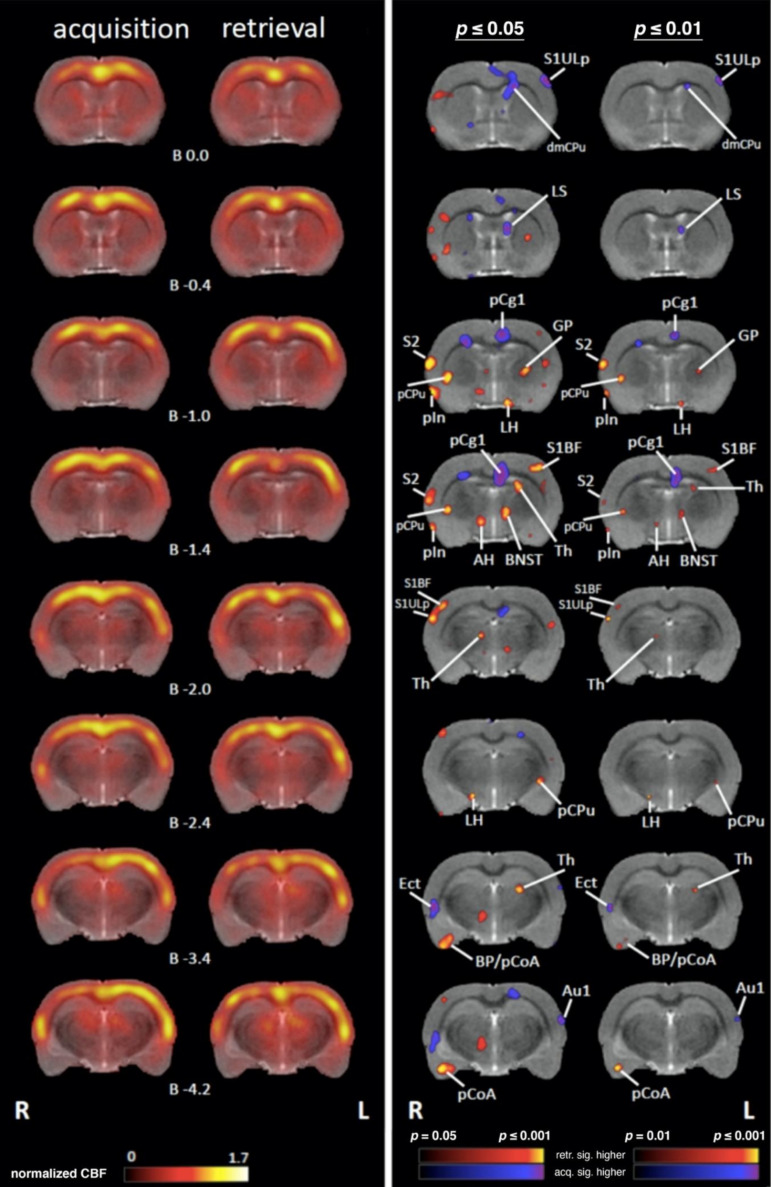

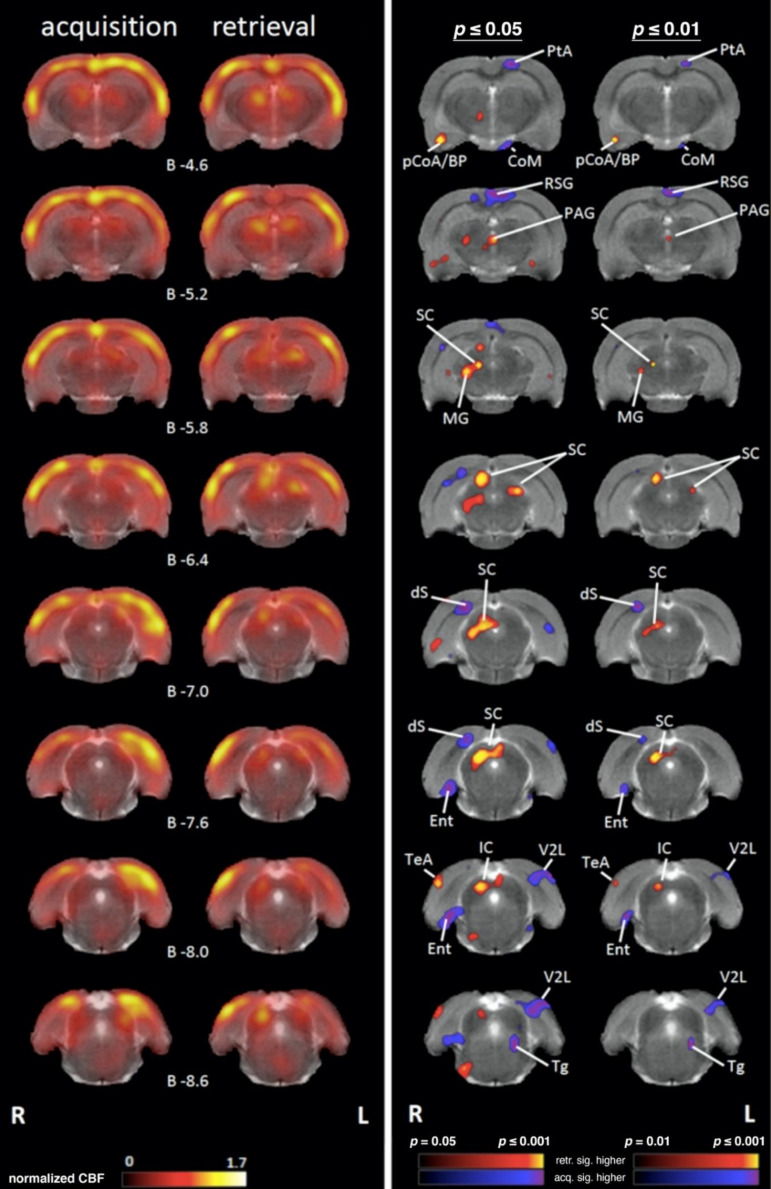
Statistically significant differences between spatial patterns of rCBF during acquisition and retrieval. Shown are maps (rostral to caudal) of normalized SPECT images of mean rCBF during acquisition and retrieval and statistically significant differences between both conditions (*n* = 10). All images are overlaid on anatomical reference MR images. Probability maps are shown with voxels at a significance level of 5% (*p* ≤ 0.05) and 1% (*p* ≤ 0.01), respectively. Voxels with significantly higher rCBF during retrieval as compared to acquisition are color-coded in red to yellow, voxels with higher rCBF during acquisition as compared to retrieval in blue to violet. The color scale for the normalized group mean flow is from 0 to 1.7 times the mean (μ) flow. The color scales for the p-maps range from *p* = 0.05 to *p* ≤ 0.001 corresponding to t-values of 2.262 to ≥4.781 for the 5% significance level and from *p* = 0.01 to *p* ≤ 0.001 corresponding to t-values of 3.250 to ≥4.781 for the 1% significance level. R, L: right or left hemisphere.

All defined brain regions for SPECT analysis were labeled according to the anatomical nomenclature of the rat brain atlas [15]. The normalized blood flow patterns compiled for all animals are illustrated in Figure 3.

Based on the individual variance in behavioral responses during the injection time on the test day, a paired t-test was performed on the brains (*n* = 10) to identify the brain pathways that were specifically activated during the acquisition and retrieval phase.

#### 4.1.1. Sensory/Motor Cortex

**Figure 4:**
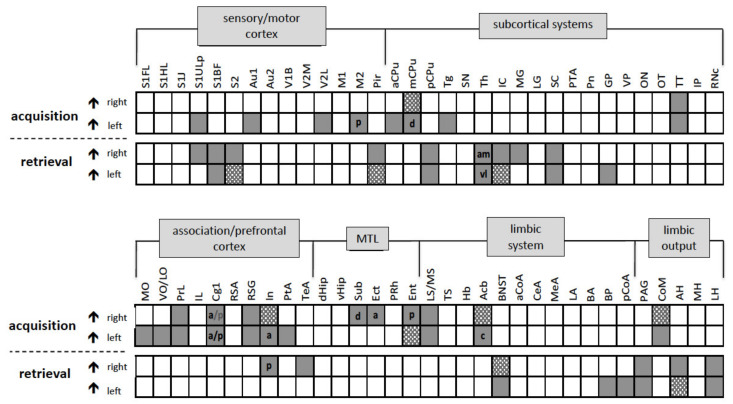
Diagrams comparing rCBF during acquisition and retrieval, respectively, within the same individual animals (*n* = 10) and schematic summary of the p-map data with a threshold level of *p* ≤ 0.01 (grey boxes) and of *p* ≤ 0.05 (hatched boxes), respectively, analyzed by paired t-tests. Brain regions are subdivided in the following systems: sensory/motor cortex, subcortical system, association-prefrontal cortex, limbic system and limbic output system. a, anterior; am, anterior medial; c, core; d, dorsal; p, posterior; sh, shell; v, ventral.

Acquisition: Regional cerebral blood flow (rCBF) was significantly higher during acquisition compared to retrieval in the left primary somatosensory cortex upper lip region (S1ULp), left primary auditory cortex, left secondary visual cortex lateral area (V2L), and left posterior secondary motor cortex (pM2) (Figure 3 and Figure 4).

Retrieval: rCBF was significantly higher during retrieval compared to acquisition in the right primary somatosensory cortex upper lip region (S1ULp) and bilaterally in the primary somatosensory cortex barrel field (S1BF) in the secondary somatosensory cortex (S2) and the piriform cortex (Pir), (Figure 3 and Figure 4).

#### 4.1.2. Subcortical Systems

Acquisition: Significantly higher rCBF was observed during acquisition compared to retrieval in the left anterior part of caudate putamen (aCPu) and the left tegmentum (Tg) and bilaterally in the medial part of caudate putamen (dmCPu) and in the tenia tecta (TT) (Figure 3 and Figure 4).

Retrieval: Significantly higher rCBF was observed during retrieval compared to acquisition in the right anterior medial (amTh) and left ventrolateral (vlTh) thalamus, in the right medial geniculate nucleus (MG), and the left globus pallidus (GP) as well as bilaterally in the inferior colliculus (IC), superior colliculus (SC), and the posterior part of the caudate putamen (pCPu) (Figure 3 and Figure 4).

#### 4.1.3. Association and Prefrontal Cortices

Acquisition: Significantly higher rCBF was observed in the left medial orbital cortex (MO), in the left ventral/lateral orbital cortex (VO/LO), and in the left parietal association cortex (PtA). Bilateral activations were observed in the in the prelimbic cortex (PrL), in the anterior and posterior cingulate cortex (a/pCg1), in the insular cortex (In), and in the granular retrosplenial cortex (RSG) during acquisition compared to retrieval (Figure 3 and Figure 4).

Retrieval: In the right posterior insular cortex (pIn) and in the right temporal association cortex (TeA), significantly higher rCBF was detected during retrieval compared to acquisition (Figure 3 and Figure 4)

#### 4.1.4. Medial Temporal Lobe (MTL)/Limbic Regions

Acquisition: Within the MTL/limbic system, significantly higher rCBF was observed in the right dorsal subiculum (dSub) and in the right anterior ectorhinal cortex (Ect) as well as bilaterally in the entorhinal cortex (Ent), lateral (LS) and medial septum (MS), and in the nucleus accumbens (Acb) during acquisition compared to retrieval (Figure 3 and Figure 4)

Retrieval: Significantly higher rCBF during retrieval compared to acquisition was observed in the left posterior basal amygdala (BP) and left posterior cortical amygdala (pCoA and bilaterally in the bed nucleus of stria terminalis (BNST) (Figure 3 and Figure 4).

#### 4.1.5. Limbic Output Regions

Acquisition: Significantly higher rCBF during acquisition compared to retrieval was observed bilaterally in the mammillary bodies (corpora mamillaria, CoM).

Only during retrieval significantly higher rCBF was found bilaterally in the periaqueductal gray (PAG) and bilaterally in the anterior hypothalamus (AH) compared to acquisition (Figure 3 and Figure 4).

Retrieval: Significantly higher rCBF during retrieval compared to acquisition was observed bilaterally in the periaqueductal grey (PAG) and in the anterior (AH) and lateral hypothalamus (LH).

## 5. Discussion

Brain-wide imaging or mapping of spatial patterns of neural activity in rodents in learning and memory paradigms is traditionally done using immediate early gene mapping techniques or autoradiographic approaches using glucose analogues [18]. These approaches can provide high spatial resolution but do not permit longitudinal imaging of the same individuals.

Only few imaging techniques are suited for brain-wide, i.e., unbiased hypothesis-free, consecutive in vivo imaging of spatial patterns of neural activity in task-performing rodents. One of these approaches is SPECT imaging of rCBF for review see [6].

CBF is a well-established proxy for neural activity. Tracer techniques for imaging CBF, including autoradiographic as well as in vivo imaging methods like SPECT and PET, have been used for more than fifty years in numerous studies for analyzing spatial patterns of neural activity in animals and humans [6]. ^99m^TcHMPAO-SPECT is similar in rationale to FDG-PET [13] but can provide higher temporal resolution and in rodents also a higher spatial resolution. During ongoing behavior, animals are injected with the lipophilic tracer ^99m^TcHMPAO. After passage through the blood-brain barrier the tracer is converted to a hydrophilic ^99m^Tc compound that is trapped in the brain [19]. The distribution of the trapped tracer can be mapped in anesthetized animals after trials for acquisition or retrieval. The distribution of the tracer represents the spatial pattern of the average rCBF during the time period of intravenous injection in the awake state.

### 5.1. Learning to Avoid an Aversive Situation—Switching from Escape to Avoidance Strategy

TWA is a complex aversive feedback learning form that humans and other animals frequently encounter in daily life [20]. In the TWA paradigm used in the present study, an individual learns to adapt to an environmental challenge (unpleasant foot shock) by developing specific behavioral strategies, an escape and later a conditioned avoidance reaction [21]. As opposed to the Pavlovian fear conditioning paradigm, the two-way active avoidance (TWA) learning form integrates both Pavlovian and instrumental components. The individual learns that an initially neutral stimulus (CS = tone) predicts an aversive event (UCS = foot shock). TWA learning can be viewed as a two-stage learning process [22] during which the individual first undergoes Pavlovian fear conditioning to form the association between two stimuli and the animal is forced to respond with an active behavioral strategy, an (innate, reflexive) escape reaction. In the second stage the individual undergoes instrumental conditioning (feedback learning) during which the initial escape or “flight” strategy is transferred into an active avoidance reaction [16,17]. However, unlike Pavlovian fear conditioning, the emergence of an active avoidance strategy requires that the animal incorporates a “prediction error” into the acquisition process [21]. The prediction error in this study was encountered during trials in which the animal incidentally moved to the “safe” compartment of the shuttle box during presentation of the CS but prior to the onset of the UCS and thereby created a situation in which the predicted foot shock did not occur. This information was most likely stored in the working memory and retrieved whenever the animal encountered a similar situation during training and eventually resulted in a goal-oriented avoidance response. An additional challenge for the animal during the acquisition phase was the conflict situation that was created by the fact that each time the animal had escaped from the UCS to the “safe” compartment of the shuttle box the following training trial required the animal to return to the “dangerous” compartment where it had encountered the UCS during the previous trial. Hence, the animal had to generalize, i.e., to understand, that safety was not assigned to a defined compartment of the shuttle box but that it was always the “other” compartment which was safe.

In line with results from our previous experiments with rats and mice [16,17,23] the animals displayed a continuous learning curve as reflected by a significant increase in avoidance responses over the five-day training period. While on the first training day (acquisition) exclusively escape reactions were observed, on the fifth test day (memory retrieval) reactions switched to conditioned avoidance responses.

The neuronal circuitry of Pavlovian fear conditioning has been extensively investigated [24,25,26,27,28]. It is clear that learning and CS-UCS association during acquisition does not occur isolated in one brain region but rather requires the concerted activation of interconnected brain circuits, including the hippocampal formation, prefrontal and orbitofrontal cortical regions, association cortex, and septum [3,18,29,30]. Our in vivo SPECT imaging study in awake freely behaving rats unveiled distinct functional neuronal networks that are specifically activated during acquisition and during memory retrieval.

### 5.2. Acquisition

#### 5.2.1. The Role of Sensory and Motor Pathways in CS-UCS Association and the Escape Strategy

The initial step on the way of associating the CS with the UCS involved hearing the tone, feeling the foot shock, and visually searching for an escape route to the “safe” compartment. Accordingly, as summarized in Figure 4, we observed higher rCBF (compared to retrieval) in the corresponding *sensory systems*, in particular subregions of the primary somatosensory cortex mediating the sensation of the electrical shock in the paws (S1HL). In addition, higher rCBF was observed in the lateral portion of the secondary visual cortex (V2L). The V2 region is a visual association area that is integrated in the ventral stream, which through its connections with the hippocampus is assumed to be involved in visual memory formation. The activation of the V2L might reflect visual orientation toward the escape route. Hearing the tone involved the activation of the primary auditory cortex (Au1), which was observed during acquisition. The auditory cortex played a key role for tone (CS) processing during the acquisition phase. Traditionally, sensory cortices have been viewed as “stimulus analyzers”, with learning and memory assigned to “higher” cortical regions [30]. However, neurophysiological studies have produced evidence for learning-induced plasticity in sensory cortices and in particular in the auditory cortex [31]. Numerous experiments in animals and human subjects provided evidence that a sound that acquires a specific behavioral relevance changes its neuronal representation in the auditory cortex (Au) for reviews, see [31,32,33]. Moreover, there is evidence that different types of sound representations may occur depending on a specific learning task, which in turn determines the behavioral meaning that a sound acquires during task performance [34]. In our experiment, a presented tone (CS) announced an unpleasant event (UCS) and—during classical and instrumental conditioning—triggered associative synaptic plasticity in the Au [21,31].

During acquisition the animal initially performed a UCS-induced escape response. This activity appears to be reflected by elevated rCBF in *motor regions,* including the motor cortex (M2), which together with regions of the caudate putamen (aCPu, mCPU) are part of the nigrostriatal system. This system is especially involved in motor planning and learning [35].

Elevated rCBF during acquisition was also observed in the *tenia tecta (TT)*, which is part of the olfactory cortex. The TT receives afferents from the mPFC [36], an area that is involved in contextual encoding and working memory, e.g., when animals move between different environmental contexts. TT neurons preferentially fire at a specific position in the trajectory of a maze during a working memory task involving odor place-matching [36]. This suggests that context-specific activity of the mPFC may contribute to the generation of context-dependent activity in the TT. The elevated rCBF that we observed in subregions of the mPFC (PrL Cg1) may support the idea that TT activity during the prediction of future behaviors might be driven via top-down inputs from higher cognitive and motivational centers in the prefrontal cortex [36].

Elevated rCBF during acquisition was also observed in the *tegmentum (Tg)*, including the nucleus ruber, which is involved in body movements, and the reticular formation, which modulates premotor arousal states, cardiovascular functions, and pain perception.

#### 5.2.2. The Role of Prefrontal/Association Cortices and Limbic Regions in CS-UCS Association, Working Memory and Avoidance Strategy

Associative learning requires the involvement of working memory, selective attention, behavioral adaptation, conflict monitoring, and conflict solving. In addition, there is evidence that negative emotional stimuli activate a wide and complex network of brain regions, including the medial prefrontal (mPFC) and anterior cingulate (ACC) cortices [37]. Such activities might be reflected by a higher rCBF during acquisition in *higher associative cortical regions*, including regions of the medial (MO, PrL, and Cg1) and lateral (VO/LO) prefrontal cortical regions, as well as in the insular cortex (In) compared to retrieval. Due to its connectivity with sensory and motor association cortices the prefrontal cortex participates in a variety of complex and highly integrated cognitive functions, such as working memory, planning, and decision-making [38,39,40]. Working memory includes processes involved in the regulatory control and maintenance of task-relevant information. As pointed out previously, throughout the acquisition trials the animals had to store various things in working memory: (a) the CS-UCS association, i.e., they had to remember that the foot shock follows the tone, (b) remember the escape route, (c) solve the conflict, i.e., learn that the UCS is not assigned to a specific compartment of the shuttle box, (d) prediction error, i.e., to learn that moving to the other compartment immediately after the tone avoids the foot shock. On the neurochemical level it was shown during in vivo micodialysis studies that dopamine release in the mPFC is essential for the formation of a goal-directed avoidance strategy [4,5].

*The a/pCg1* regions displayed significantly higher rCBF during acquisition compared to retrieval. The *pCg1* is involved in cognitive control and mediating ongoing adaptive behavior (in our experiment, to transfer the escape strategy into an avoidance strategy) [41,42,43]. Similar to findings in humans, animal experiments revealed that the *aCg1* is part of a broad network that might be involved in the early stages of learning, during which effort, flexibility, and behavioral adaptation are important. Human studies revealed the aCg1 is involved in error awareness [44], which is an important step during avoidance learning. Furthermore, the aCg1has been shown to be involved in value encoding, decision making, previous reactions, and outcomes [45]. In addition, the aCg1 plays an essential role in conflict and performance monitoring (i.e., in our experiment to move to the compartment where the foot shock was encountered during the previous trial) and the anticipation of emotionally related reward or punishment [37,46]. Finally, elevated rCBF in Cg1 during acquisition might reflect its involvement in the regulation of various autonomic functions related to stress responses, including changes in blood pressure and heart rate [47].

Various studies suggest that the aCg1 and the *insular cortex (In)* are involved in error awareness and recognition and the initiation of adaptive responses to error and negative feedback [48,49,50]. In addition, there is evidence that these cortical regions are major components of the system for the flexible control of goal-directed behavior [51]. During acquisition the In displayed elevated rCBF compared to the retrieval phase. The In is reciprocally connected with frontal cortical regions including the aCg1, the orbitofrontal, and the medial prefrontal cortices, and also has various connections with regions of the limbic system. These includes afferents from the lateral and basolateral amygdala; efferents to the basolateral, lateral and central amygdala nuclei; and connections with the BNST, the mediodorsal nucleus of the thalamus, the lateral hypothalamus, and parahippocampal regions [50]. Due to its widespread prefronto-limbic connections the In can be viewed as an integration hub conferring various sensory, emotional, motivational, and cognitive functions [50] and linking them to motor output systems. An fMRI study in human subjects [52] provided evidence for the hypothesis that the In is responsible for preparing for the sensory and affective impact of touch (e.g., in our learning paradigm the sensation of the foot shock). Hence, the activation of the In during acquisition may reflect the experience of fear and anxiety [50], which is a key feature of this learning phase. This is also supported by recent studies in rodents revealing that the In is involved in the modulation of anxiety-like behavior with distinct regional differences: rostral regions have an anxiogenic role, while medial and caudal regions have an anxiolytic role [53]. Finally, higher rCBF in the In may also reflect cognitive and motivational decision-making aspects [54] in order to avoid punishment and maximize reward (by avoiding the foot shock).

The *ventral (VO), lateral (LO) and medial (MO) areas of the orbitofrontal cortex (OFC)* displayed significantly elevated rCBF during acquisition compared to retrieval. The OFC is involved in cognitive learning tasks and working memory and it was shown in a fear conditioning study in humans that the activity of the OFC was sustained throughout the acquisition phase [55]. The OFC is involved in stimulus evaluation [56,57], e.g., in our experiment the emotionally relevant situation evoked by the aversive unconditioned stimulus (foot shock). The OFC was also shown to be involved in encoding prediction errors [58,59], which is an essential prerequisite for shifting from escape to avoidance behavior. The increase in blood flow activity in the MO and (unilaterally) in the VO/LO during acquisition may be indicative of such processes, including those related to attention, decision making, and emotional evaluation [54,55]. Moreover, the elevated rCBF in the MO observed in our SPECT study may support the hypothesis that avoiding an aversive outcome is rewarding. This is supported by an fMRI study in humans using an aversive feedback task that showed enhanced activity in the MO during avoidance, indicating that this reflects an intrinsic reward signal that serves to reinforce avoidance behavior [60].

During acquisition we also observed elevated rCBF in *limbic brain regions (Acb, LS/MS, dSub, aEct, pEnt)*. The involvement of *the lateral septum (LS)* during the acquisition of avoidance learning was shown in our imaging study in rats using the ^14^C-2-fluoro-deoxyglucose imaging technique, revealing elevated metabolic activity in the lateral septum (LS) during acquisition compared to habituation (novelty) and retrieval [18], which is in accordance with observations of the present study. In general, the septum is important in fear learning and the lateral septum is crucial for processing of the CS-UCS association [61].

Higher rCBF was also observed in the *nucleus accumbens (Acb)* during acquisition compared to retrieval. The abundance of cAcb afferents from limbic regions and strong Acb efferents to motor regions indicate that it may serve as an interface between the limbic and motor systems [62,63]. The Acb is considered to be involved in the cognitive processing of motor functions associated with reward and reinforcement and in the encoding of new motor programs that facilitate the acquisition of a given reward [64]. Microinfusion studies revealed that the Acb is essential for the CS-UCS association [65] and it was shown that the Acb receives projections from the amygdala, which is essentially involved in discriminative cue selectivity [66]. Furthermore, the Acb appears to be involved in signal predictions related to the anticipated foot shock [67] and thereby contributes to the computation of prediction errors (probably signaled by dopamine neurons), which are critical for reinforcement learning [68]. Finally, the Acb has been discussed to mediate Pavlovian-instrumental transfer [69]. During acquisition the animal learned to associate a sound (CS) with an unpleasant stimulus (UCS). During ongoing training the Pavlovian sound-foot shock association learned during the acquisition phase was transferred to the instrumental situation, i.e., the avoidance response, a behavioral strategy that removed the animal from the aversive stimulus with which it was paired.

Higher rCBF during acquisition compared to retrieval was observed in the *distal subiculum (dSub)*, the major hippocampal output structure, transferring spatial information to its various cortical/subcortical areas, including the nucleus accumbens (Acb) [70], (which also showed elevated rCBF during acquisition (see above)). It was recently shown that subicular neurons encode multiple types of navigation-associated information and thereby transfer “how”, “which”, and “when” information to downstream target regions [70] and that disconnection of the Sub–Acb pathway impairs spatial working memory, as observed in a radial arm maze task [71].

Higher rCBF during acquisition compared to retrieval was observed in the *entorhinal cortex (Ent)*, the interface between the hippocampal formation and the neocortex. The Ent confers information about directions in the environment [72], which during the acquisition phase of TWA learning is important to learn about the escape and avoidance route.

### 5.3. Retrieval

#### The Role of Prefrontal, Subcortical Sensory and Limbic Systems in Memory Recall and Behavioral Strategy

With respect to prefrontal regions, elevated rCBF was observed during retrieval in the insular cortex (In) and in the temporal association cortex (TeA). A recent study in mice provided evidence that the *insular cortex (In)* is crucially involved in processing and modulating aversive emotions and exerts top-down regulation of ongoing behavior [73]. Other studies in rodents support a role for the In in mediating emotional behavior, including learned fear [74,75,76,77] as well as in mediating approach versus avoidance behavior [78]. It was shown in rats [79] and humans [80] that the posterior part of the In (pIn) contains distinct auditory (CS) and somatotopically organized somatosensory fields (UCS) with an overlapping “associative” region in which these sensory modalities are integrated. It was also shown in rodents that the In has a role in the modulation of anxiety-like behavior [53].

Our SPECT study revealed higher rCBF in the *caudal temporal association cortex (TeA)* during retrieval compared to acquisition. Rodent studies suggest that TeA connectivity resembles those of the temporal association cortex in primates. Multitracing experiments revealed heterogeneous zones within the TeA along the rostro-caudal axis: the rostral TeA is bidirectionally connected with oro-facial areas, the middle TeA shares connectivity with somatosensory-motor areas as well as with visual and auditory areas [81,82,83], and the caudal TeA, i.e., the TeA subregion activated during retrieval in our SPECT study, connects with medial prefrontal areas and the ventral hippocampus [84]. These connectivities suggest a role of the TeA in essential components of TWA learning in particular during memory retrieval, including sensory perception and contextual memory associated with emotion, and, as behavioral studies suggest, in linking representations of sensory stimuli and predicted outcomes.

During retrieval significantly elevated rCBF was observed in *subcortical sensory* regions compared to acquisition. *Auditory subcortical regions (IC, MG)* displayed elevated rCBF during retrieval compared to acquisition, which might be related to decision making, i.e., to avoid the foot shock, as stated in the Weinberger model [85]. In the Weinberger model the tone information is processed from the cochlea through the inferior colliculus (IC) and the ventral medial geniculate body (MGv) to reach the auditory cortex. The tone information also reaches the medial part of the medial geniculate body (MGm) where it converges with the UCS information. The MGm response is projected to the amygdala, which controls the conditioned response, i.e., the avoidance reaction. There is additional evidence that subcortical projections of the IC and MG mediate emotional responses conditioned to acoustic stimuli [86]. The MG projects to a number of other subcortical regions involved in emotional behavior, such as the lateral amygdala (LA) and hypothalamus (AH) [86], i.e., both regions which in our SPECT study also displayed elevated rCBF during retrieval compared to acquisition.

During retrieval the *visual superior colliculus (SC)* also displayed higher rCBF compared to acquisition, which may be indicative of subcortical visual activity while performing the avoidance response, compared to higher rCBF during acquisition in the cortical V2 region compared to retrieval. This transition from visual cortical activity during acquisition to subcortical visual activity during retrieval may indicate that once the direction to the “safe” compartment was learned, the activation of the associative visual cortex V2 is no longer required.

With respect to limbic/MTL pathways we observed higher rCBF during retrieval in regions that are part of the “extended amygdala”, i.e., subnuclei of the amygdala (BP, pCoA) and the BNST. Elevated rCBF during retrieval was observed in the *posterior basal (BP)* and *posterior cortical amygdala (pCoA)* compared to acquisition. These observations are somewhat contradictory to findings in rabbits (using a wheel-running avoidance paradigm) where the amygdala appeared to be particularly important during the acquisition of instrumental avoidance behavior but did not impair avoidance responding [87]. However, various studies provide evidence that the amygdala plays a central role in fear conditioning and CS-UCS association region [3,88,89], which suggests that the amygdala is involved in the expression of fear memory retrieval.

Higher CBF observed in the BP during retrieval compared to acquisition may reflect the consolidation (and perhaps also retrieval) of fear-cued memory, which might be mediated through its connections with the subiculum (Sub) and the pCoA. The pCoA also displayed elevated rCBF during retrieval compared to acquisition, which may reflect its involvement in modulating memory processing via its connections to the medial temporal lobe memory system [90].

During retrieval significantly higher rCBF was observed in the *bed nucleus of the stria terminalis (BNST)* compared to acquisition. Based on its connections with the amygdala, dorsal raphe, hippocampus, hypothalamus, medulla, nucleus accumbens, periaqueductal gray, and prefrontal cortical regions, the BNST is considered to act as an interface between the “affective forebrain” (including the amygdala, ventral hippocampus, and medial prefrontal cortex) and the hypothalamic and brainstem areas that mediates neuroendocrine, autonomic, and behavioral responses to actual or anticipated threats [91]. The BNST was proposed to mediate conditioned defensive responses in relation to the temporal predictability of a CS when an aversive outcome will occur [91], which is an essential step during the transition of an escape to an avoidance response.

During retrieval higher rCBF was observed in the *periaqueductal grey (PAG)* compared to acquisition. This is in line with the view that the PAG is a key center of controlling defensive behaviors and that it plays a critical role in motivated behavior and behavioral responses to threatening stimuli [92,93,94]. In addition, there is also evidence that the PAG plays a role in the assessment of prediction errors [95], which is an essential component of avoidance learning.

## 6. Conclusions

**Figure 5:**
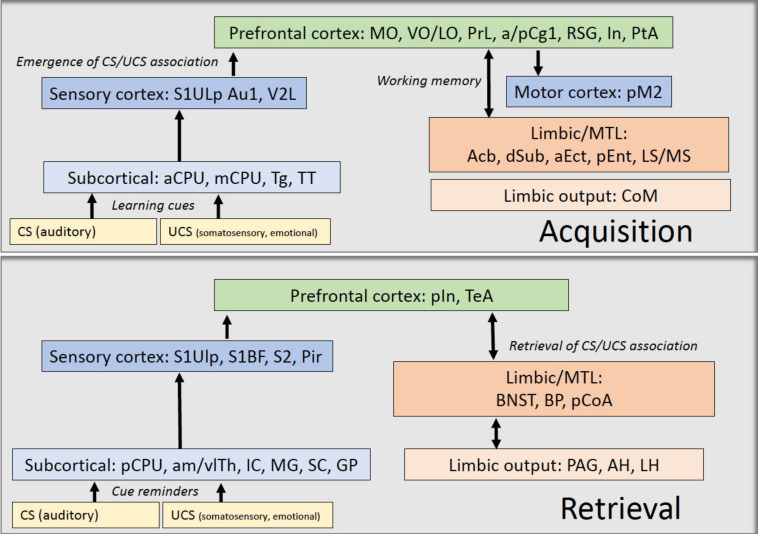
Hypothetical pathways compiled from imaging data during acquisition and during retrieval of TWA learning. Color coding: dark blue, sensory/motor cortex; light blue, subcortical regions; green, prefrontal cortical areas; dark red, limbic/MTL areas; light red, limbic output regions. For abbreviations, see list of abbreviations.

Taken together, as summarized in Figure 5, we were able to identify distinct neuronal circuits specifically recruited during the acquisition and retrieval phases of a two-way avoidance learning task. During acquisition more prefrontal cortical and associative cortical regions displayed elevated rCBF compared to retrieval, which most likely indicates that prior to the emergence of the association between the CS and UCS the animal activates regions involved in working memory in its attempt “to make sense” of the novel learning situation and to figure out an appropriate behavioral strategy. In addition, more limbic/MTL brain regions displayed elevated rCBF during acquisition compared to retrieval, which on the one hand might reflect the modulation of stress levels (In) and on the other hand may indicate that the recruitment of limbic brain regions—in cooperation with prefrontal association regions—reflects working memory functions. With respect to sensory pathways, the elevated rCBF in cortical (Au1, V2L) areas observed during acquisition compared to retrieval may reflect the recruitment of higher order sensory processing in order to “understand” the relevance of the CS (Au1) and to encode visual cues (V2L) of the escape (and later avoidance) route. This interpretation might also be supported by the elevated rCBF observed in the hippocampal input and output regions (dSub, Ent), indicating the processing and integration of spatial information to various cortical and subcortical areas, including prefrontal cortical regions and the nucleus accumbens (Acb), that also showed elevated rCBF during acquisition.

Once the animal has achieved a successful, goal-directed avoidance strategy, the sensory cortical activation appears to be “transferred” to subcortical sensory regions (IC, MG, SC), which might—once the avoidance strategy has been learned—be “sufficient” for the execution of the conditioned behavioral response. The activation of the In may reflect downregulation of anxiety induced by aversive memory and a top-down monitoring of the ongoing behavioral strategy. Higher rCBF in the TeA during retrieval may reflect the integration of sensory perception and contextual memory and the association of the representations of the sensory stimuli with predicted outcomes (i.e., avoiding the foot shock). With respect to limbic/MTL regions the higher rCBF observed in the BNST during retrieval may indicate the processing of the temporal predictability of the CS of the aversive outcome (UCS) and the memorization of the conditioned avoidance response. Higher rCBF in the PAG may reflect the control of conditioned defensive behavior (avoidance) in response to a threatening stimulus (CS). Activations in the posterior basal amygdala (BP) and the posterior cortical amygdala (pCoA) during retrieval compared to acquisition may reflect the modulation, consolidation, and perhaps also retrieval of fear-cued memory processing.

## Glossary

Acb	nucleus accumbens
shAcb	n. accumbens—shell region
cAcb	n. accumbens—core region
AH	anterior hypothalamus
Au1	primary auditory cortex
Au2	secondary auditory cortex
BA	anterior basal amygdala
BNST	bed nucleus of the stria terminalis
BP	posterior basal amygdala
CeA	central amygdala
Cg1	cingular cortex
CoA	cortical amygdala
CoM	corpus mammillare
CPu	caudate putamen
Ect	ectorhinal cortex
Ent	entorhinal cortex
GP	globus pallidus
Hip	hippocampus
IC	inferior colliculus
IL	infralimbic cortex
aIn	anterior insular cortex
pIn	posterior insular cortex
IP	interpeduncular nucleus
LA	lateral amygdala
LG	lateral geniculatum
LH	lateral hypothalamus
LS	lateral septum
M1	primary motor cortex
M2	secondary motor cortex
MeA	medial amygdala
MG	medial geniculatum
MH	medial hypothalamus
MO	medial orbitofrontal cortex
MS	medial septum
MTL	medial temporal lobe
ON	olfactory nucleus
OT	olfactory tubercle
PAG	periaqueductal grey
Pir	piriform cortex
Pn	pontine nucleus
PRh	perirhinal cortex
PrL	prelimbic cortex
PTA	area pretectalis
PtA	parietal association cortex
RNc	raphe nuclei
RSA	agranular retrosplenial cortex
RSG	granular retrosplenial cortex
S1BF	primary somatosensory cortex—*barrel cortex*
S1FL	primary somatosensory cortex—*forelimb*s
S1HL	primary somatosensory cortex—*hindlimb*s
S1J	primary somatosensory cortex—*jaw region*
S1ULp	primary somatosensory cortex—*upper lip*
S2	secondary somatosensory cortex
SC	superior colliculus
SF	septofimbrial nucleus
SG	subgeniculatum
SN	substantia nigra
Sub	subiculum
TeA	temporal association cortex
Tg	tegmentum
Th	thalamus
TS	triangular septum
TT	taenia tecta
V1B	primary visual cortex—*binocular area*
V1M	primary visual cortex—*monocular area*
V2L	secondary visual cortex—lateral
V2M	secondary visual cortex—medial
VG	ventral geniculatum
VO/LO	ventral/lateral orbitofrontal cortex
VP	ventral pallidum
VTA	ventral tegmental area
